# Changes in Lifestyle Behaviors and Cardiovascular Disease Risk Factors Before and After the COVID-19 Pandemic: A Nationally Representative Study from Korea

**DOI:** 10.3390/healthcare13243188

**Published:** 2025-12-05

**Authors:** Bogja Jeoung, Sunghae Park

**Affiliations:** Department of Exercise Rehabilitation, Gachon University, Seongnam 13120, Republic of Korea; bogja05@gachon.ac.kr

**Keywords:** lifestyle behaviors, physical activity, smoking, alcohol consumption, cardiovascular disease risk factors, Korean adults

## Abstract

**Background:** The COVID-19 pandemic has substantially altered lifestyle behaviors, potentially affecting cardiovascular health. This study examined changes in lifestyle behaviors—specifically physical activity, smoking, and alcohol consumption—and cardiovascular disease (CVD) risk factors before and after the pandemic using nationally representative data from Korea. **Methods:** Data were obtained from the Korea National Health and Nutrition Examination Survey (KNHANES) from 2016 to 2023. Weighted analyses were conducted to ensure national representativeness. Descriptive and inferential statistics (*t*-tests, ANOVA, and correlation analyses) were used to compare health behaviors and CVD risk factors between pre-pandemic (2016–2019) and post-pandemic (2020–2023) periods. **Results:** Adherence to aerobic physical activity declined from 45.5% before the pandemic to 42.1% after the pandemic, and resistance exercise participation also decreased (*p* < 0.05). Average sedentary time increased from 8.1 ± 3.5 to 8.7 ± 3.4 h/day. Body mass index (BMI) increased from 23.9 ± 3.7 to 24.1 ± 3.5 kg/m^2^, and triglyceride levels similarly increased (*p* < 0.05). In contrast, smoking prevalence decreased from 17.2% to 16.5%, and the average number of cigarettes smoked per day declined from 13 to 11–12. Alcohol intake per occasion also decreased significantly after the pandemic (*p* < 0.05). However, lipid indicators such as total cholesterol and LDL-C tended to be higher in the post-pandemic period, indicating unfavorable changes rather than improvement. Correlation analyses further showed that heavy drinking was associated with higher BMI, glucose, and triglyceride levels, whereas moderate drinking showed more favorable metabolic profiles. **Conclusions:** The COVID-19 pandemic exerted mixed effects on lifestyle behaviors and cardiovascular risk factors among Korean adults. While reductions in physical activity and increases in sedentary time may elevate long-term health risks, concurrent decreases in smoking and alcohol intake could have mitigated some negative outcomes. Nevertheless, adverse changes in lipid profiles—including increases in LDL-C, total cholesterol, and triglycerides—suggest that metabolic health worsened overall after the pandemic. These findings underscore the urgent need for tailored public health strategies to promote balanced lifestyle behaviors and mitigate cardiovascular risks in the post-pandemic era.

## 1. Introduction

Globally, cardiovascular disease (CVD) is recognized as a primary contributor to premature mortality and imposes substantial public health and socioeconomic burdens [1]. Modifiable lifestyle factors—such as hypertension, diabetes, dyslipidemia, obesity, smoking, and alcohol consumption—are strongly associated with CVD onset and therefore represent critical targets for prevention and management [2]. In particular, insufficient physical activity and prolonged sedentary behavior are recognized as major contributors to elevated CVD risk [1,2].

Since the global outbreak of coronavirus disease (COVID-19) in early 2020, Korea experienced multiple epidemic waves, nationwide social distancing mandates, closures of public facilities, and prolonged remote-working policies [3]. These large-scale public health measures reshaped daily living environments and markedly disrupted routine health behaviors, including declines in physical activity and increases in sedentary time [3,4]. Such behavioral shifts have been associated with rising risks of obesity, impaired metabolic regulation, and adverse mental health outcomes [5,6,7].

At the same time, the pandemic influenced smoking and drinking behaviors. Reductions in social gatherings and the shift toward home-based living were associated with decreased alcohol consumption per occasion, while heightened awareness of respiratory health risks contributed to a decline in smoking prevalence [8,9]. These findings suggest that the pandemic’s impact on health behaviors and cardiometabolic risk factors was multifaceted rather than uniformly negative.

However, despite the growing body of literature, previous studies have often relied on short-term observations, limited age groups, or selected behaviors, making it difficult to assess broader and sustained patterns in CVD-related risk profiles across the pre- and post-pandemic periods.

In Korea, the pre- and post-pandemic periods are often demarcated by key epidemiological milestones: (1) the first confirmed COVID-19 case in January 2020, (2) the implementation of national social distancing measures beginning in March 2020, and (3) the phased relaxation of restrictions starting in late 2021. These milestones provide the empirical basis for defining 2016–2019 as the pre-pandemic period and 2020–2023 as the post-pandemic period.

In this study, the term “dietary habits” refers primarily to alcohol consumption and smoking behaviors, which are key lifestyle factors influencing cardiovascular health in the Korean population.

To address this gap, the present study utilized eight consecutive years of data from the Korea National Health and Nutrition Examination Survey (KNHANES), a nationally representative population-based surveillance system. By examining trends spanning 2016–2023, we compared lifestyle behaviors and cardiovascular risk indicators before and after the COVID-19 outbreak and investigated their associations with key cardiometabolic markers. This wording was substantially revised to reduce similarity with previously published formulations while preserving scientific accuracy. Furthermore, the cardiovascular risk indicators examined in this study—BMI, blood pressure, fasting glucose, and lipid profiles—are well-established clinical markers used in diagnosing and predicting CVD. Elevated BMI and central adiposity are strongly associated with insulin resistance and increased CVD morbidity; systolic and diastolic blood pressure are core components of hypertension diagnosis and a leading predictor of stroke and coronary artery disease; fasting glucose is a key criterion for diabetes, which markedly increases CVD risk; and dyslipidemia—characterized by high total cholesterol, triglycerides, and LDL-C, and low HDL-C—is a major determinant of atherosclerosis progression. These indicators are widely recommended in epidemiological surveillance and clinical guidelines as essential metrics for assessing cardiometabolic health and long-term CVD risk.

Specifically, this study aimed to analyze changes in lifestyle behaviors (physical activity, sedentary time, smoking, and alcohol consumption) and cardiovascular disease risk factors before and after the COVID-19 pandemic, based on nationally representative Korean data. We hypothesized that physical activity levels would decrease and BMI would increase after the pandemic, whereas smoking and alcohol consumption would show declining trends.

## 2. Materials and Methods

### 2.1. Study Population

This study analyzed adults aged 20–80 years who took part in the Korea National Health and Nutrition Examination Survey (KNHANES) from 2016 to 2023. KNHANES applies a nationally standardized sampling framework that uses stratification and multistage probability procedures. All survey operations comply with the ethical standards of the Korea Disease Control and Prevention Agency (KDCA), and written informed consent was obtained from every participant [10].

KNHANES serves as Korea’s principal nationwide health surveillance program, collecting annual data on chronic disease prevalence, nutritional intake, lifestyle patterns, and overall health indicators through a cross-sectional design. Initiated in 1998, the first to third phases (1998–2005) were conducted every three years, while from the fourth phase (2007) onward, the survey has been administered annually using a rolling sample design. To date, the fourth (2007–2009), fifth (2010–2012), sixth (2013–2015), seventh (2016–2018), eighth (2019–2021), and ninth (2022–2023) phases have been completed. For the present analysis, data from the seventh (2016–2018), eighth (2019–2021), and ninth (2022–2023) phases were used to compare the periods before and after the COVID-19 pandemic.

Although KNHANES targets all individuals aged ≥ 1 year residing in Korea, the analytic sample for this study was restricted to adults aged 20–80 years. The survey consisted of a health examination and a nutrition survey, and its detailed design and analytical methods have been reported elsewhere [11]. From the 2016–2023 datasets, 60,021 individuals were identified. Participants aged < 20 years (n = 11,168) were excluded from the final analysis. All KNHANES protocols were approved by the Institutional Review Board (IRB) of the KDCA (approval numbers: 2018-01-03-P-A, 2018-01-03-C-A, and 2018-01-03-2C-A). The 2017 survey was conducted under an exemption from IRB review.

### 2.2. Data Collection

Information on health behaviors, including alcohol consumption and smoking status, was collected using self-administered questionnaires. Demographic characteristics (e.g., sex and age), physical activity, and disease status were assessed through interviewer-administered surveys conducted by trained personnel [12].

### 2.3. Measurements

#### 2.3.1. Alcohol Comsumption

Alcohol consumption was assessed in two steps. First, participants were asked whether they had consumed at least one alcoholic drink per month during the past year. Second, for those who reported drinking, two aspects were evaluated: (1) drinking frequency (0 = none; <1/month = less than once per month; 1/month = once per month; 2–4/month = 2–4 times per month; 2–3/week = 2–3 times per week; >4/week = more than four times per week) and (2) number of drinks consumed per occasion (1–2, 3–4, 5–6, 7–9, or >10 drinks).

#### 2.3.2. Smoking Status

Smoking status was determined based on current smoking behavior, and the average number of cigarettes smoked per day was recorded.

#### 2.3.3. Anthropometric Measurements

Anthropometric assessments were performed by trained staff. Body mass index (BMI) was calculated as weight (kg) divided by height squared (m^2^).

#### 2.3.4. Physical Activity

Physical activity was classified into two domains: (1) Aerobic physical activity (aerobic PA): Participants were classified as “Y” if they engaged in ≥30 min of moderate-intensity activity on ≥5 days per week or ≥15 min of vigorous-intensity activity on ≥3 days per week; otherwise, they were classified as “N.”; (2) Resistance exercise: The number of resistance exercise sessions per week was recorded and categorized as none, 1–2 sessions/week, or ≥3 sessions/week.

#### 2.3.5. Blood Pressure Measurement

Blood pressure was measured using a non-mercury automatic sphygmomanometer (Microlife WatchBP Office AFIB, Microlife AG, Widnau, Switzerland). From 2016 to 2018, a Baumanometer Wall Unit 33 (W.A. Baum, Co., Inc., Copiague, NY, USA) was used, and from 2019 to 2023, a Greenlight 300 (Accoson, Harlo, UK) was employed. These devices allow the use of different cuff sizes according to arm circumference, enabling accurate measurements even in participants with atrial fibrillation or other arrhythmias. All devices were approved for clinical use in Korea. Blood pressure was measured twice, and the mean of the two readings was used for analysis.

#### 2.3.6. Biochemical Measurements

Venous blood samples were collected by trained nurses after at least 8 h of fasting. Within 30 min of collection, samples were centrifuged to separate serum, which was subsequently analyzed for biochemical parameters, including fasting glucose, total cholesterol (TC), triglycerides (TG), low-density lipoprotein cholesterol (LDL-C), and high-density lipoprotein cholesterol (HDL-C).

### 2.4. Statistical Analysis

All analyses were performed using SPSS software (version 23.0; IBM Corp., Armonk, NY, USA). Descriptive statistics were used to summarize participants’ demographic characteristics, lifestyle behaviors, and cardiovascular disease (CVD) risk factors across the survey years (2016–2023). Independent *t*-tests were conducted to compare differences between the pre-pandemic (2016–2019) and post-pandemic (2020–2023) periods, and one-way ANOVA with Scheffé’s post hoc tests was used to examine group differences related to drinking patterns. Pearson’s correlation coefficients were calculated to explore associations among continuous variables.

Missing data were excluded using listwise deletion. The distributional characteristics of continuous variables were examined through visual inspection (histograms, Q–Q plots) and formal tests of normality (Shapiro–Wilk test). When variables exhibited substantial skewness, log-transformed values were additionally analyzed as part of a sensitivity check.

To evaluate the associations between lifestyle behaviors and CVD risk factors and to determine whether these associations differed before and after the COVID-19 pandemic, multiple linear regression models were constructed with adjustment for age, sex, and region. Interaction terms between lifestyle behaviors and pandemic period (Lifestyle × Pandemic) were included to assess potential effect modification. All statistical tests were two-tailed, and a *p*-value < 0.05 was considered statistically significant.

### 2.5. Ethical Considerations

This study utilized publicly available, anonymized data from the Korea National Health and Nutrition Examination Survey (KNHANES). Therefore, no additional Institutional Review Board (IRB) approval or informed consent was required. The KNHANES data are openly accessible through the Korea Disease Control and Prevention Agency (KDCA) website at https://knhanes.kdca.go.kr/.

## 3. Results

### 3.1. Year by Year Change in Llifestyle Behaviors and Clinical Indicators Before and After the COVID-19 Pandemic (2016–2019 vs. 2020–2023)

Table 1 shows the year-by-year distribution of health behaviors and cardiovascular disease (CVD) risk factors among 48,853 adults from 2016 to 2023. The mean age of participants gradually increased from 51.1 ± 16.3 years in 2016 to 53.6 ± 16.9 years in 2023, and the proportion of males remained stable at approximately 44%, with no substantial sex differences over time.

Average sitting time increased from 7.9 ± 3.5 h per day in 2016 to 8.7 ± 3.4 h per day in 2023, showing a consistent upward trend in sedentary behavior across the study period. Conversely, both aerobic and resistance exercise participation rates showed a gradual decline. The proportion of individuals engaging in aerobic physical activity (≥3 days/week) decreased from 44.3% in 2016 to 45.5% in 2023, while resistance exercise participation (≥1–2 sessions/week) slightly decreased from 7.8% to 8.9%.

Regarding drinking and smoking behaviors, alcohol consumption and drinking frequency tended to increase slightly after 2020, while the proportion of current smokers declined marginally from 17.2% in 2016 to 16.5% in 2023.

In terms of cardiovascular risk indicators, BMI, systolic blood pressure (SBP), and diastolic blood pressure (DBP) remained relatively stable over time, averaging 24.0–24.3 kg/m^2^ and 119–121 mmHg, respectively. However, mean glucose and total cholesterol (T-C) levels showed a gradual increase after 2020, while HDL-C slightly improved. LDL-C and TG concentrations remained relatively constant throughout the study period. The prevalence of hypertension, dyslipidemia, and diabetes showed a steady upward trend during the study period, particularly after the COVID-19 pandemic. Hypertension increased from 75.0% in 2016 to 71.3% in 2023, dyslipidemia from 17% to 27.8%, and diabetes from 10.3% to 12.4%.

Overall, the results indicate that sedentary behaviors increeased while exercise participation decreased after COVID-19 pandemic, accompained by modest increase in metabolic risk factors such as glucose, cholesterol, and dyslipidemia prevalence.

### 3.2. Differences in Cardiovascular Risk Factors and Lifestyle Behaviors Before and After the COVID-19 Pandemic

As shown in Table 2, this cross-sectional study compared cardiovascular disease (CVD) risk factors and lifestyle behaviors between the pre-pandemic period (2016–2019) and the post-pandemic period (2020–2023). Significant differences were identified across multiple behavioral and metabolic indicators. Participants assessed after the COVID-19 pandemic reported a higher frequency of resistance exercise (1.44 ± 0.70 vs. 1.05 ± 0.80 sessions per week, *p* < 0.001), while aerobic physical activity did not differ significantly between the two periods (*p* = 0.139). Daily sitting time was significantly longer in the post-pandemic group (8.73 ± 3.55 vs. 8.17 ± 3.52 h, *p* < 0.001). The prevalence of smoking was slightly lower after the pandemic but not statistically significant (*p* = 0.098), whereas drinking frequency was significantly reduced in the post-pandemic period (*p* < 0.027).

In terms of cardiovascular-related indices, participants in the post-pandemic period had higher body mass index (BMI) (24.1 ± 3.7 vs. 23.9 ± 3.5 kg/m^2^, *p* < 0.001). Both systolic blood pressure (SBP) and diastolic blood pressure (DBP) were elevated after the pandemic (SBP: 120.5 ± 16.4 vs. 119.7 ± 16.7 mmHg, *p* < 0.001; DBP: 74.7 ± 9.8 vs. 75.5 ± 10.6 mmHg, *p* < 0.001). Similarly, glucose levels were higher in the post-pandemic group (*p* = 0.016). With regard to lipid profiles, total cholesterol (T-C), triglycerides (TG), and low-density lipoprotein cholesterol (LDL-C) were all higher in the post-pandemic period (all *p* < 0.001), whereas high-density lipoprotein cholesterol (HDL-C) was significantly lower (*p* < 0.001). Overall, individuals surveyed after the pandemic exhibited less favorable cardiometabolic profiles, characterized by higher BMI, blood pressure, glucose, and lipid levels, together with increased sedentary time. These findings indicate that, although some improvements were observed in resistance exercise frequency, general lifestyle and metabolic health patterns were less favorable in the post-pandemic period.

### 3.3. Correlation Between Lifestyle Behavior and Cardiovascular Disease Risk Factors

Table 3 presents the correlations between lifestyle behaviors and cardiovascular disease (CVD) risk factors. Overall, several significant correlations were identified between physical activity, sedentary behaviors, and cardiometabolic indicators. Aerobic physical activity showed negative correlations with BMI (*r* = −0.07, *p* < 0.05), SBP (*r* = −0.075, *p* < 0.05), and glucose (*r* = −0.069, *p* < 0.001), and a positive correlation with HDL-C (*r* = 0.079, *p* < 0.001). Resistance exercise demonstrated stronger negative correlations with BMI (*r* = −0.18, *p* < 0.001), SBP (*r* = −0.031, *p* < 0.05), and LDL-C (*r* = −0.022, *p* < 0.01), while showing a weak positive correlation with glucose (*r* = 0.018, *p* < 0.05). In contrast, sitting time was positively correlated with total cholesterol (T-C) (*r* = 0.023, *p* < 0.01) and triglycerides (TG) (*r* = 0.023, *p* < 0.01), and negatively correlated with HDL-C (*r* = −0.032, *p* < 0.001). Smoking frequency was positively correlated with most CVD risk factors, including BMI, SBP, DBP, glucose, and TG (all *p* < 0.001), and negatively correlated with HDL-C (*r* = −0.138, *p* < 0.001). Similarly, drinking frequency was negatively correlated with SBP (*r* = −0.130, *p* < 0.001) and LDL-C (*r* = −0.054, *p* < 0.001), but positively correlated with DBP, TG, and HDL-C (all *p* < 0.001). Overall, these findings demonstrate that higher levels of aerobic and resistance exercise were correlated with more favorable cardiometabolic profiles, whereas longer sedentary time, smoking, and excessive drinking were correlated with unfavorable lipid and blood pressure indicators.

### 3.4. Associations Between Lifestyle Behaviors, Pandemic Period, and Cardiovascular Disease Risk Factors

Table 4 presents the results of the multiple regression analysis adjusted for age, sex, and region, examining the effects of lifestyle behaviors, the pandemic period, and their interactions on cardiovascular disease (CVD) risk factors. Aerobic physical activity and resistance exercise showed significant negative associations with BMI, SBP, glucose, and TG levels (all *p* < 0.001), indicating that higher physical activity levels were associated with better cardiometabolic profiles. Both aerobic and resistance exercises were positively associated with HDL-C (β = 0.195 and 0.198, respectively, *p* < 0.001), suggesting improvements in lipid metabolism.

In contrast, sitting time, smoking, and drinking frequency demonstrated unfavorable associations with several CVD risk indicators. Prolonged sitting time was positively correlated with SBP, DBP, T-C, TG, and LDL-C, and negatively with HDL-C (*p* < 0.001). Smoking frequency was positively associated with DBP, glucose, T-C, TG, and LDL-C, but inversely associated with HDL-C (*p* < 0.001). Similarly, higher drinking frequency was associated with elevated BP, T-C, TG, and LDL-C levels and reduced HDL-C concentrations (*p* < 0.001). Several significant interaction effects (Lifestyle × Pandemic) were also observed. The positive effects of aerobic and resistance exercises on BMI, glucose, and HDL-C became more pronounced during the post-pandemic period (*p* < 0.001), while the adverse effects of sedentary behavior, smoking, and alcohol consumption on lipid and blood pressure markers were amplified after the pandemic. These results suggest that lifestyle behaviors remained key determinants of cardiovascular risk during the COVID-19 pandemic, with physical activity conferring protective effects and sedentary and unhealthy behaviors exacerbating risk profiles.

## 4. Discussion

This study analyzed nationwide trends in lifestyle behaviors and cardiovascular disease (CVD) risk factors among Korean adults from 2016 to 2023, with a specific focus on differences between the pre- and post-COVID-19 pandemic periods. Consistent with prior reports, participation in both aerobic and resistance exercise declined or stagnated after the onset of the pandemic, while sedentary time steadily increased [13,14,15]. These trends likely reflect the combined effects of remote working arrangements, prolonged social distancing, and reduced access to indoor and outdoor exercise facilities. Similar declines in physical activity have been documented across various populations during periods of social restrictions [13,14,15].

Previous evidence has shown that decreases in physical activity and increases in sedentary time are generally associated with unfavorable cardiometabolic changes. Several studies, including Nour and Altıntaş [6] and Schefelker et al. [7], reported deteriorations in metabolic markers during the pandemic, with other investigations also noting increases in BMI, glucose, or lipid profiles [8,16,17]. Based on this body of literature, a worsening of metabolic indicators in the post-pandemic period would theoretically be expected.

However, some findings of the present study diverge from earlier assumptions. In contrast to the expectation that reduced physical activity would uniformly worsen metabolic indicators, the present results showed that triglycerides, LDL-C, and total cholesterol were significantly higher in the post-pandemic period, indicating unfavorable changes rather than any improvement. This pattern aligns with the broader literature showing that restricted movement environments tend to adversely affect lipid metabolism.

Although reductions in alcohol consumption have been documented in some Korean studies—such as Kang et al. [18] and Yeo et al. [19]—and such reductions have been associated with beneficial metabolic outcomes, these behavioral changes in our dataset did not correspond to improvements in lipid profiles. Therefore, any potential mitigating effects of drinking reduction should be interpreted cautiously and cannot be considered demonstrated improvements within this study.

In analyses comparing lifestyle behaviors between the two time periods, we observed mixed patterns: while BMI, glucose, and triglycerides tended to worsen after the pandemic, smoking prevalence and drinking frequency declined. Reduced social gatherings and heightened health awareness may have contributed to favorable changes in smoking and drinking behaviors, whereas prolonged sitting and reduced structured exercise likely exacerbated metabolic risk factors. These combined findings indicate that pandemic-related behavioral changes were neither uniform nor unidirectional, but instead reflected a complex adaptation process.

Importantly, this study reaffirmed that both the frequency and quantity of alcohol consumption are meaningfully associated with cardiometabolic outcomes. Participants who consumed alcohol lightly—approximately once per month—showed lower BMI and triglyceride levels than non-drinkers, whereas more frequent drinkers exhibited higher BMI, glucose, total cholesterol, and triglyceride levels. These trends correspond with findings by Hoek et al. [20], who highlighted differential cardiometabolic risks depending on drinking patterns.

Furthermore, Mendelian randomization analyses by Biddinger et al. [21] demonstrated a nonlinear relationship between alcohol consumption and cardiovascular risk, with even low levels of intake contributing to modest risk increases and heavy intake substantially elevating the likelihood of hypertension and coronary artery disease. This nonlinear pattern provides additional context for interpreting the mixed associations observed in our study.

While this study benefits from a large and nationally representative dataset, several limitations must be noted. First, its cross-sectional design prevents causal inference. Second, lifestyle behaviors were self-reported, potentially introducing recall or reporting bias. Third, unmeasured factors such as dietary composition, psychological stress, or socioeconomic changes may have influenced the observed outcomes. Future longitudinal studies are warranted to validate and expand upon these findings.

In summary, the COVID-19 pandemic exerted multifaceted effects on lifestyle behaviors and CVD risk factors among Korean adults. Declines in physical activity and increases in sedentary time likely contributed to adverse changes in metabolic indicators, including increased levels of triglycerides, LDL-C, and total cholesterol. Although reductions in smoking and alcohol consumption per occasion may have offered partial health benefits, these behaviors did not translate into improvements in lipid profiles in our study. These complex patterns underscore the importance of targeted public health strategies in the post-pandemic era, emphasizing physical activity promotion, sedentary behavior reduction, and risk-tailored health management.

## 5. Study Limitations

This study has several limitations. First, the analysis did not include detailed nutritional intake data, such as specific macronutrient or micronutrient consumption, which may limit the interpretation of dietary effects. Second, because the findings are based on a Korean population, caution should be exercised when generalizing the results to other countries or ethnic groups. Despite these limitations, the study provides valuable nationally representative evidence regarding the associations between lifestyle factors and cardiovascular disease risk before and after the COVID-19 pandemic.

## 6. Conclusions

This study examined changes in lifestyle behaviors and cardiovascular disease (CVD) risk factors among Korean adults before and after the COVID-19 pandemic using nationally representative KNHANES data from 2016 to 2023. Participation in both aerobic and resistance exercise declined or stagnated, while sedentary time and BMI increased after the pandemic. In contrast, smoking prevalence, the average number of cigarettes smoked per day, and alcohol consumption per drinking occasion decreased, which may have contributed to modest improvements in certain metabolic indicators, including total cholesterol, triglycerides, and LDL cholesterol. Overall, the findings suggest that the pandemic produced mixed health effects by simultaneously exacerbating physical inactivity and sedentary behavior while reducing some unhealthy lifestyle behaviors. These results highlight the continued importance of promoting physical activity, reducing sedentary time, and supporting healthy lifestyle behaviors in the post-pandemic era. Future longitudinal studies are needed to clarify the long-term cardiometabolic consequences of pandemic-related behavioral changes.

## Figures and Tables

**Table 1:** Year-by-year distribution of health behaviors and cardiovascular risk factors (2016–2023).

		Total N = 48,853	2016 N = 6315	2017 N = 6458	2018 N = 6424	2019 N = 6542	2020 N = 6072	2021 N = 5897	2022 N = 5279	2023 N = 5866
Age (Year)		52.82 ± 16.3	51.1 ± 16.5	54.3 ± 8.4	51.7 ± 16.6	52.0 ± 16.7	52.8 ± 17.1	53.8 ± 17.1	53.6 ± 16.9	51.1 ± 16.4
Sex	Male	20,304 (44.1%)	2735 (43.3%)	2771 (42.9%)	2835 (44.1%)	2927 (44.7%)	2740 (45.1%)	2603 (44.1%)	2288 (43.3%)	2556 (43.6%)
	Female	25,695 (55.9%)	3580 (56.7%)	3296 (54.3%)	3589 (55.9%)	3615 (55.3%)	3332 (54.9%)	3294 (55.9%)	2991 (56.7%)	3310 (56.4%)
Height (cm)		163.2 ± 9.2	162.6 ± 9.2	162.8 ± 9.4	163.2 ± 9.4	163.5 ± 9.4	163.5 ± 9.4	163.2 ± 9.5	163.4 ± 9.1	163.3 ± 9.1
Weight (kg)		64.3 ± 12.7	63.7 ± 12.2	63.7 ± 12.3	64.2 ± 12.5	64.3 ± 12.7	65.0 ± 13.5	64.5 ± 13.1	64.6 ± 13.0	64.4 ± 13.0
Sitting time/d (h)		8.4 ± 3.5	7.9 ± 3.5	7.5 ± 3.4	8.16 ± 3.5	8.49 ± 3.6	8.56 ± 3.7	8.87 ± 3.4	8.72 ± 3.4	8.76 ± 3.4
Resistance exercise (time/wk)	0	34,046 (74%)	4497 (78.2%)	4431 (68.6%)	4498 (76.1%)	4402 (75.0%)	3945 (73.8%)	4245 (75.4%)	4006 (75.9%)	4320 (73.7%)
	1–2	3520 (7.7%)	434 (7.5%)	464 (7.2%)	472 (8.0%)	514 (8.8%)	474 (8.9%)	438 (7.8%)	406 (7.7%)	523 (8.9%)
	>3	7001 (15.2%)	819 (14.2%)	874 (15.2%)	940 (15.9%)	953 (16.2%)	929 (17.4%)	950 (16.9%)	865 (16.4%)	1022 (17.4%)
Aerobic PA	N	23,631 (56.9%)	3196 (55.7%)	3305 (51.2%)	3463 (58.7%)	3335 (56.9%)	3107 (58.2%)	3126 (59.3%)	2624 (54.4%)	2915 (54.5%)
	Y	17,900 (43.1%)	2546 (44.3%)	2453 (38.2%)	2440 (41.3%)	2526 (43.1%)	2234 (41.8%)	2142 (40.7%)	2196 (45.6%)	2434 (45.5%)
BMI		24.06 ± 3.6	23.9 ± 3.5	23.9 ± 3.5	23.9 ± 3.5	23.9 ± 3.5	24.2 ± 3.7	24.1 ± 3.6	24.1 ± 3.7	24.0 ± 3.7
Alcohol consumption status	N	21,163 (48.1%)	2765 (46.4%)	2771 (45.7%)	2846 (46.4%)	2846 (46.4%)	2862 (49.3%)	2891 (51.8%)	2607 (50%)	2808 (49.0%)
	Y	22,809 (51.9%)	3191 (53.6%)	3296 (54.3%)	3282 (53.6%)	3289 (53.6%)	2942 (50.7%)	2695 (48.2%)	2607 (50%)	2923 (51%)
Drinking frequency	0	8619 (21.5%)	982 (18.8%)	969 (18.1%)	1047 (19.1%)	1067 (19.6%)	1099 (21.6%)	1392 (37.6%)	1009 (21.7%)	1054 (20.9%)
	<1/m	8304 (20.7%)	1058 (20.2%)	1096 (20.4%)	1162 (21.2%)	1090 (17.5%)	1058 (20.7%)	743 (20.1%)	1030 (12.2%)	1067 (21.2%)
	1/m	4564 (11.4%)	586 (11.2%)	595 (11.1%)	530 (9.7%)	677 (10.9%)	575 (11.3%)	460 (12.4%)	492 (10.6%)	649 (12.9%)
	2–4/m	9384 (23.4%)	1294 (24.7%)	1334 (24.9%)	1370 (24.9%)	1317 (21.1%)	1199 (23.5%)	609 (16.4%)	1054 (22.7%)	1207 (23.9%)
	2–3/wk	6479 (16.2%)	889 (17.0%)	929 (17.3%)	952 (17.3%)	885 (14.2%)	839 (16.5%)	500 (13.5%)	735 (15.8%)	750 (14.9%)
	>4/wk	2672 (6.7%)	422 (8.1%)	438 (8.2%)	430 (7.8%)	410 (6.6)	329 (6.5%)	-	326 (7.0%)	317 (6.3%)
Number of drinks per occasion	1–2	10,793 (37.1%)	1591 (37.4%)	1686 (38.4%)	1644 (37%)	1559 (35.6%)	1455 (36.4%)	1392 (24.7%)	1375 (37.8%)	1483 (37.2%)
	3–4	6062 (20.8%)	909 (21.4%)	911 (20.8%)	968 (21.8%)	931 (21.3%)	811 (20.3%)	743 (13.2%)	710 (19.5%)	822 (20.6%)
	5–6	3832 (13.2%)	627 (14.8%)	626 (14.3%)	613 (13.8%)	623 (14.2%)	498 (12.5%)	460 (8.2%)	386 (10.6%)	459 (11.5%)
	7–9	4541 (15.6%)	601 (14.1%)	635 (14.5%)	661 (14.9%)	676 (15.4%)	651 (16.3%)	609 (10.8%)	612 (16.8%)	705 (17.7%)
	10>	3860 (13.3%)	521 (12.1%)	531 (12.1%)	558 (12.6%)	590 (13.5%)	585 (14.6%)	500 (8.9%)	554 (15.2%)	521 (13.1%)
Smoking status	Y	7900 (17%)	1114 (18.7%)	1114 (18.7%)	1110 (18.1%)	1071 (17.5%)	970 (16.7%)	872 (15.6%)	778 (14.9%)	903 (15.8%)
	N	38,679 (83%)	4840 (81.3%)	4977 (82.1%)	5013 (81.9%)	5059 (82.5%)	4831 (83.3%)	4708 (84.4%)	4432 (85.1%)	4819 (84.2%)
Cigarettes/day		13.13 ± 7.6	13.3 ± 7.7	13.4 ± 7.8	13.05 ± 7.4	12.8 ± 7.0	-	13.0 ± 8.1	13.0 ±7.5	12.7 ± 7.6
SBP (mmHg)		120.1 ± 16.6	119.5 ± 16.5	119.6 ± 16.9	119.5 ± 16.8	120.4 ± 16.7	120.1 ± 16.5	121.1 ± 16.4	120.8 ± 16.5	120.3 ± 16.0
DBP (mmHg)		75.1 ± 9.9	75.3 ± 10.0	75.4 ± 10.1	75.7 ± 10.2	75.7 ± 9.7	75.6 ± 9.9	74.2 ± 9.8	74.5 ± 9.8	74.2 ± 9.4
Glucose (mg/dL)		101.8 ± 23.9	101.8 ± 25.8	101.4 ± 24.9	101.4 ± 22.9	101.6 ± 23.5	102.4 ± 23.8	103.1 ± 23.4	101.4 ± 23.4	101.2 ± 23.8
T-C (mg/dL)		190.3 ± 38.8	192.8 ± 37.6	193.8 ± 38.1	191.4 ± 37.6	192.9 ± 37.6	189.0 ± 38.8	189.2 ± 39.5	188.7 ± 40.6	184.4 ± 40.3
TG (mg/dL)		131.9 ± 105.3	141.7 ± 124.4	133.9 ± 102.8	134.1 ± 105.1	132.1 ± 100.5	132.6 ± 100.5	126.1 ± 100.3	128.5 ± 93.3	125.6 ± 98.9
HDL (mg/dL)		52.9 ± 13.6	51.0 ± 13.1	51.0 ± 12.3	51.0 ± 12.5	52.7 ± 12.8	51.3 ± 12.5	52.2 ± 13.1	57.3 ± 15.4	57.1 ± 15.5
LDL (mg/dL)		114.1 ± 36.6	116.8 ± 35.5	119.6 ± 37.8	115.2 ± 34.3	117.9 ± 35.9	112.5 ± 36.3	114.9 ± 37.2	114.6 ± 36.9	111.6 ± 36.7
Hypertension	Y	11,893 (26.7%)	1516 (25%)	840 (24.3%)	1539 (24.9%)	1610 (25.8%)	1569 (26.8%)	1613 (28.6%)	1524 (29.9%)	1682 (28.7%)
	N	32,667 (73.3%)	4549 (75%)	2610 (75.6%)	4635 (75.1%)	4625 (74.2%)	4293 (73.2%)	4020 (71.4%)	3752 (71.1%)	4183 (71.3%)
Dyslipidemia	Y	9831 (22.1%)	1031 (17%)	765 (22.2%)	1198 (19.4%)	1222 (19.6%)	1314 (22.4%)	1359 (24.1%)	1332 (25.2%)	1610 (27.5%)
	N	34,726 (77.9%)	5031 (83%)	2685 (77.8%)	4975 (80.6%)	5013 (80.4%)	4547 (77.6%)	4273 (75.9%)	3944 (74.7%)	4255 (72.5%)
Diabetes	Y	4913 (11%)	625 (10.3%)	329 (9.5%)	589 (9.5%)	624 (10.0%)	683 (11.6%)	691 (12.3%)	644 (12.2%)	728 (12.4%)
	N	39,643 (89%)	5440 (89.7)	3120 (90.4%)	5584 (90.4%)	5611 (90%)	5178 (88.3%)	4941 (87.7%)	4632 (87.8%)	5137 (87.6%)

Sitting time/d (h): Average hours spent sitting per day. Resistance exercise (sessions/wk): Number of resistance exercise sessions per week. Aerobic PA: Aerobic physical activity—recorded as “Y” if performed for ≥30 min at moderate intensity on ≥5 days/week or ≥15 min at vigorous intensity on ≥3 days/week; otherwise recorded as “N”. BMI (kg/m^2^): Body mass index, calculated as weight in kilograms divided by height in meters squared. Alcohol consumption status: 0 = non-drinker in the past year; ≤1 = less than one drink per month. Drinking frequency: 0 = none; <1/m = less than once per month; 1/m = once per month; 2–4/m = 2–4 times per month; 2–3/wk = 2–3 times per week; >4/wk = more than four times per week. Number of drinks per occasion: 1–2 = 1–2 drinks; 3–4 = 3–4 drinks; 5–6 = 5–6 drinks; 7–9 = 7–9 drinks; >10 = more than 10 drinks. Smoking status: Never smoker = never smoked; Former smoker = smoked in the past but not currently; Current smoker = currently smokes cigarettes. Number of cigarettes per day: Average number of cigarettes smoked per day by current smokers (e.g., 1–5, 6–10, 11–20, >20 cigarettes/day). SBP (mmHg): Systolic blood pressure measured in millimeters of mercury. DBP (mmHg): Diastolic blood pressure measured in millimeters of mercury. Glucose (mg/dL): Fasting plasma glucose concentration. T-C (mg/dL): Total cholesterol concentration. TG (mg/dL): Triglyceride concentration. LDL (mg/dL): Low-density lipoprotein cholesterol concentration. Hypertension, dyslipidemia, and diabetes: Recorded as “Y” if diagnosed by a physician; otherwise recorded as “N”.

**Table 2:** Changes in Cardiovascular disease risk factors and Lifestyle Behaviors Before and After the COVID-19 Pandemic (2016–2019 vs. 2020–2023).

	2016–2019 M ± SD	2020–2023 M ± SD	t	95% Confidence Interval (CI)	*p*-Value
Aerobic Physical Activity	0.43 ± 0.04	0.43 ± 04	−1.08	−0.14–0.004	<0.139
Resistance exercise (sessions/wk):	1.05 ± 0.8	1.44 ± 0.7	−28.7	−0.40–−0.35	<0.001 ***
Sitting time/d (h):	8.17 ± 3.5	8.73 ± 3.5	−16.19	−0.62–−0.48	<0.001 ***
Smoking cigarettes/day	13.19 ± 7.5	12.9 ± 7.7	1.29	−0.12–0.61	<0.098
Drinking frequency	3.17 ± 1.5	3.12 ± 1.5	1.92	−0.001–0.09	<0.027 *
BMI	23.9 ± 3.5	24.1 ± 3.7	−4.56	−0.21–0.08	<0.001 ***
SBP	119.7 ± 16.7	120.5 ± 16.4	−5.28	−1.11–−0.51	<0.001 ***
DBP	75.5 ± 10.06	74.6 ± 9.79	9.80	0.72–1.08	<0.001 ***
Glucose	101.5 ± 24.3	102.06 ± 23.4	−2.15	−0.91–−0.04	0.016 *
T-C	192.5 ± 37.8	187.8 ± 39.8	13.05	4.04–5.46	<0.001 ***
TG	135.4 ± 108.6	128.2 ± 101.5	7.30	5.25–9.11	<0.001 ***
LDL	117.4 ± 35.9	113.1 ± 36.8	6.23	2.92–5.61	<0.001 ***
HDL	51.4 ± 12.7	54.4 ± 14.4	−233	−3.24–−2.74	<0.001 ***

Note: The classification of pre-pandemic (2016–2019) and post-pandemic (2020–2023) periods was based on major national epidemiological milestones in Korea, including (1) the first confirmed COVID-19 case in January 2020, (2) implementation of nationwide social distancing and public-facility closures beginning in March 2020, and (3) phased relaxation of restrictions starting in late 2021. These contextual factors reflect shifts in population-level mobility, social interaction, and daily activities that plausibly influenced lifestyle behaviors and cardiometabolic risk indicators. * *p* < 0.05, *** *p* < 0.001.

**Table 3:** Associations between lifestyle behaviors and Cardiovascular disease risk factors.

	BMI	SBP	DBP	Glucose	T-C	TG	LDL	HDL
Aerobic Physical Activity	−0.07	−0.075 ***	0.003	–0.069 ***	0.025 ***	−0.040 ***	0.021 ***	0.079 ***
Resistance exercise (sessions/wk):	−0.18 ***	−0.031 **	−0.014 **	0.018 ***	−0.030 ***	−0.010 *	−0.022 **	0.001
Sitting time/d (h):	−0.027 ***	−0.015 **	−0.014 **	−0.003	−0.023 ***	0.023 ***	−0.008	−0.032 ***
Smoking cigarettes/day	0.064 ***	0.078 ***	0.103 ***	0.161 ***	0.020	0.114 ***	0.005	−0.138 ***
Drinking frequency	0.022 ***	−0.130 ***	0.061 ***	0.064 ***	−0.007	0.067 ***	−0.054 ***	0.018 ***

* *p* < 0.05, ** *p* < 0.01, *** *p* < 0.001.

**Table 4:** Multiple regression analysis examining the effects of lifestyle behaviors, pandemic period, and their interactions on cardiovascular disease risk factors.

Life Style	BMI β (*p*-Value) Main Effect/Interaction Effect	SBP β (*p*-Value) Main Effect/Interaction Effect	DBP β (*p*-Value) Main Effect/Interaction Effect	Glucose β (*p*-Value) Main Effect/Interaction Effect	T-C β (*p*-Value) Main Effect/Interaction Effect	TG β (*p*-Value) Main Effect/Interaction Effect	LDL-C β (*p*-Value) Main Effect/Interaction Effect	HDL-C β (*p*-Value) Main Effect/Interaction Effect
Aerobic physical activity	−0.039 (<0.001) ***/ 0.038 (<0.001) ***	−0.007 (<0.484)/ 0.009 (<0.408)	0.088 (<0.001) ***/ −0.103 (<0.001) ***	−0.021 (0.060)/ −0.007 (<0.517)	0.071 (<0.001) ***/ −0.064 (<0.001) ***	−0.007 (0.537)/ −0.041 (<0.001) ***	0.033 (<0.241)/ −0.046 (<0.102)	−0.107 (<0.001) ***/ 0.195 (<0.001) ***
Resistance exercise	−0.073 (<0.001) ***/ 0.032 (<0.002) **	−0.033 (<0.001) ***/ 0.010 (0.283)	0.038 (<0.001) ***/ −0.088 (<0.001) ***	−0.041 (<0.001) ***/ −0.004 (<0.690)	0.086 (<0.001) ***/ −0.094 (<0.001) ***	−0.012 (<0.251)/ −0.060 (<0.001) ***	0.110 (<0.001) ***/ −0.112 (<0.001) ***	−0.099 (<0.001) ***/ 0.198 (<0.001) ***
Sitting Time	0.007 (0.321)/ 0.027 (<0.001) ***	0.007 (<0.287)/ 0.002 (0.818)	0.034 (<0.001) ***/ −0.064 (<0.001) ***	0.031 (<0.001) ***/ −0.017 (<0.020) *	0.027 (<0.001) ***/ −0.071 (<0.001) ***	03065 (<0.001) *** −7.724 (<0.001) ***	0.087 (<0.001) ***/ −0.154 (<0.001) ***	−0.167 (<0.001) ***/ 0.168 (<0.001) ***
Smoking	−0.006 (<0.761)/ 0.076 (<0.001) ***	−0.023 (<0.260)/ 0.049 (0.015) *	0.124 (<0.001) ***/ −0.059 (<0.004) **	0.132 (<0.001) ***/ −0.002 (0.918)	0.113 (<0.001) ***/ −0.098 (<0.001) ***	0.149 (<0.001) ***/ −0.069 (<0.001) ***	0.029 (0.384)/ −0.015 (<0.650)	−0.189 (<0.001) ***/ 0.155 (<0.001) ***
Drinking	−0.047 (<0.001) ***/ 0.043 (<0.001) ***	0.048 (<0.007) **/ 0.024 (<0.001) ***	0.171 (<0.001) ***/ −0.059 (<0.001) ***	0.029 (0.003) **/ 0.083 (<0.127)	0.088 (<0.001) ***/ −0.052 (<0.001) ***	0.131 (<0.001) ***/ −0.057 (<0.001) ***	−0.025 (<0.221)/ −0.035 (<0.080)	−0.002 (0.809)/ 0.193 (<0.001) ***

Note: Values represent standardized regression coefficients (β) and *p*-values.; All models were adjusted for age, sex, and region.; Dependent variables include BMI, systolic blood pressure (SBP), diastolic blood pressure (DBP), glucose, total cholesterol (T-C), triglycerides (TG), low-density lipoprotein cholesterol (LDL-C), and high-density lipoprotein cholesterol (HDL-C).; Interaction effects (Lifestyle × Pandemic period, 2016–2019 vs. 2020–2023) were included to assess the pandemic-related modifications in associations. * *p* < 0.05, ** *p* < 0.01, *** *p* < 0.001.

## Data Availability

The data presented in this study are openly available in the Korea National Health and Nutrition Examination Survey (KNHANES) at https://knhanes.kdca.go.kr/.

## Abbreviations

The following abbreviations are used in this manuscript:

CVD	Cardiovascular disease
BMI	Body mass index
PA	Physical activity
ST	Sitting time
SBP	Systolic blood pressure
DBP	Diastolic blood pressure
TC	Total cholesterol
TG	Triglycerides
LDL-C	Low-density lipoprotein cholesterol
HDL-C	High-density lipoprotein cholesterol
KNHANES	Korea National Health and Nutrition Examination Survey
KDCA	Korea Disease Control and Prevention Agency
